# A Multi-Scale Approach to Model K^+^ Permeation Through the KcsA Channel

**DOI:** 10.3389/fmolb.2022.880660

**Published:** 2022-07-08

**Authors:** T. L. Horng, R. S. Chen, M. V. Leonardi, F. Franciolini, L. Catacuzzeno

**Affiliations:** ^1^ Department of Applied Mathematics, Feng Chia University, Taichung, Taiwan; ^2^ Department of Life Science, Tunghai University, Taichung, Taiwan; ^3^ Department of Chemistry, Biology and Biotechnology, University of Perugia, Perugia, Italy

**Keywords:** K channels, permeation, KcsA, molecular dynamics, Bikerman–Poisson–Boltzmann, kinetic model, IV curve

## Abstract

K^+^ channels allow a very efficient passage of K^+^ ions through the membrane while excluding Na^+^ ions, and these properties are essential for life. The 3D structure of the KcsA K^+^ channel, solved more than 20 years ago, allows to address many relevant aspects of K^+^ permeation and selectivity mechanisms at the molecular level. Recent crystallographic data and molecular dynamics (MD) studies suggest that no water is normally present inside the selectivity filter (SF), which can instead accommodate four adjacent K^+^ ions. Using a multi-scale approach, whereby information taken from a low-level simulation approach is used to feed a high-level model, we studied the mechanism of K^+^ permeation through KcsA channels. More specifically, we used MD to find stable ion configurations under physiological conditions. They were characterized by two adjacent K^+^ ions occupying the more central positions of the SF (sites S2 and S3), while the other two K^+^ ions could be found at the external and internal entrances to the SF. Sites S1 and S4 were instead not occupied by K^+^. A continuum Bikerman–Poisson–Boltzmann model that takes into account the volume of the ions and their dehydration when entering the SF fully confirmed the MD results, showing peaks of K^+^ occupancy at S2, S3, and the external and internal entrances, with S1 and S4 sites being virtually never occupied by K^+^. Inspired by the newly found ion configuration in the SF at equilibrium, we developed a simple kinetic permeation model which, fed with kinetic rate constants assessed from molecular meta-dynamics, reproduced the main permeation properties of the KcsA channel found experimentally, including sublinear current-voltage and saturating conductance-concentration relationships. This good agreement with the experimental data also implies that the ion configuration in the SF we identified at equilibrium would also be a key configuration during permeation.

## Statement of Significance

K^+^ channels allow a very efficient passage of K^+^ ions through the membrane while excluding Na^+^ ions, and these properties are essential for life. We studied these mechanisms of K^+^ permeation through KcsA channels using a multi-scale approach, whereby information taken from low-level simulations is used to feed a high-level model. More specifically, we developed a simple kinetic permeation model which, fed with kinetic rate constants assessed from molecular meta-dynamics, reproduced the main permeation properties of the KcsA channel found experimentally.

## Introduction

K^+^ channels are membrane proteins that allow a very efficient passage of K^+^ ions while excluding Na^+^ ions. They are essential for the establishment of the negative resting potential across the membrane and the repolarization phase of the action potential. The bacterial KcsA channel has been amongst the most studied K^+^ channels, and the first to have its structure solved by X-ray crystallography (Doyle et al., 1998). Soon after the resolution of the crystal structure, the group of Christopher Miller performed electrophysiology experiments on KcsA channels (Lemasurier et al., 2001) and found properties quite similar to those previously found for many mammalian K^+^ channels, such as sublinear current-voltage (IV) relationship under symmetrical K^+^ conditions, outward rectification (i.e., the channel conducts better in the outward direction), and saturating conductance-concentration relationship. Thus, any plausible mechanism of K^+^ permeation through the KcsA channel should explain and reproduce these features observed experimentally.

Structurally, the KcsA channel is formed by the juxtaposition of four identical protein subunits, each composed of two transmembrane segments, TM1 and TM2 (Doyle et al., 1998). Starting from the cytoplasmic side (the bundle crossing), the four TM2s of the KcsA channel form the lining of the water-filled internal cavity, which is ∼10 Å wide and extends into the lipid membrane for about two-thirds of its thickness. At the extracellular pore entrance, the four P loops from each subunit form a narrow selectivity filter (SF) that is about 12 Å long and 3 Å wide and allows the passage of only naked ions (without their hydration shell). The SF is formed by a highly conserved sequence of amino acids (TVGYG) that have their carbonyl (hydroxyl in the case of threonine) oxygens pointing toward the center of the pore. Since these oxygens have a partial negative charge, their electrostatic interactions with the permeating K^+^ ions likely contribute to the permeation process.

Our comprehension of the permeation properties of KcsA moved greatly forward when the high-resolution electron density maps allowed us to clearly identify four high electron density positions inside the selectivity filter (called sites S1 to S4, from extracellular to intracellular). Two other electron-dense positions were found: one immediately outside the extracellular entrance (site S0) and the other below S4, at the center of the intracellular cavity (site Scav) (Morais-Cabral et al., 2001). Unfortunately, at that time, it was not possible to determine whether the observed electron densities inside the selectivity filter originated from K^+^ ions or from water, as they give a very similar X-ray diffraction pattern. A number of observations and physicochemical considerations initially suggested that the four binding sites (S1–S4) within the SF were alternatingly occupied by two K^+^ ions—sitting either in S1 and S3 or in S2 and S4—and two water molecules in the remaining sites (Bernèche and Roux, 2001; Zhou and MacKinnon, 2003).

Later experiments, based on anomalous diffraction X-ray crystallography and solid-state nuclear magnetic resonance, pointed instead to a selectivity filter exclusively occupied by K^+^ ions (Langan et al., 2018; Öster et al., 2019). In accordance, in long MD simulations that allowed to observe thousands of K^+^ permeation events, K^+^ ions were frequently found simultaneously occupying the sites S2 and S3, and the passage of water molecules through the SF was virtually never seen (Furini et al., 2009; Köpfer et al., 2014; Wu, 2017).

In either case, permeation would occur with a K^+^ ion approaching the SF from one side and pushing the single file of K^+^ ions (and possibly water molecules) forward to the other side (Morais-Cabral et al., 2001). This mechanism is usually referred to as knock-on (“soft” or “hard”, depending on the presence or absence of water), meaning that the incoming K^+^ ion pushes forward the single file of whatever is in the SF. Interestingly, Kratochvil and colleagues (Kratochvil et al., 2016) made MD simulations with the SF displaying either KWKW (K-water-K-water; the soft knock-on mechanism) or 0KK0 (void-K-K-void; the hard knock-on mechanism) configuration. Their results showed that only an SF in the soft knock-on configuration could predict two-dimensional infrared (2D IR) spectra that matched experimental data from isotope-labeled semi-synthetic KcsA channels and were thus interpreted as evidence against the hard knock-on mechanism. However, a follow-up study using MD simulations found that both mechanisms can generate 2D IR spectra compatible with the experimental data (Kopec et al., 2018). This study in fact indicated that 2D IR spectroscopy cannot effectively distinguish between these two conduction mechanisms in K^+^ channels.

Unfortunately, none of the mechanisms proposed until now, based on structural considerations and MD results, has been tested for its ability to correctly predict the channel behavior in terms of current-voltage and conductance-concentration relationships. This is because if on one side MD simulations have revealed atomic details of the binding sites and the energetics in the SF, on the other side, they can hardly predict the shape of the IV relationships under different experimental conditions. Even very long simulations can greatly underestimate the channel conductance (i.e., by nearly 40 folds at a voltage of 300 mV as in Jensen et al., 2013). Also, the outcome of other approaches, such as Brownian dynamics (BD) simulation (Bernèche and Roux, 2003) and Poisson–Nernst–Planck (PNP) type models (Furini et al., 2006; Dyrka et al., 2013; Liu and Lu, 2017), employed to compute the IV curves, was rather unsuccessful in predicting the experimental data (Lemasurier et al., 2001). Taking all this together, it appears that more information and new approaches are required to determine the exact mechanism of K^+^ permeation through the SF, and more key physical properties need to be included in the modeling.

To find more quantitative and predictive mechanisms of permeation, kinetic models could be used in conjunction with MD simulations in a multi-scale approach. Kinetic models picture the channel pore in a few stable configurations, and the permeation process as ions hopping from one stable configuration to the next, with an associated probability given by the rate constant characterizing that transition (Hille, 2001). Although kinetic models can only give an approximate picture of the permeation process, they are able to connect the model output to experimental results through the flux equations that can easily be obtained from the model. This will allow to evaluate the soundness of a postulated permeation process derived from structural data and MD results. One such model has been recently found using long MD simulations, where K^+^ permeation through the KcsA channel can be described by a quite simple sequence of Markov states (Domene et al., 2021). In this type of model, which we may define as an association/dissociation model (A/D model, (Nelson, 2011)), the current is produced by the binding of a K^+^ ion to the SF on one side of the membrane and the unbinding of another K^+^ ion on the other side. In this case, the exit (unbinding) of the K^+^ ion is not to be seen as directly linked to (in fact, it is temporally separated from) the entry of a K^+^ ion on the other side. Notably, in contrast to the classical knock-on mechanism (Hodgkin and Keynes, 1955), these types of models do predict sublinear IV relationships and saturating current-concentration curves (Nelson, 2011), in line with experimental data.

Based on these considerations, in this study, we tested whether an A/D type permeation model could reproduce the experimentally observed permeation properties from KcsA channels. The approach used in this study was: 1) to exploit MD simulations and structure-based Poisson-Boltzmann (PB) modeling (modified to include steric and dehydration effects) for sketching the A/D reaction scheme of K^+^ permeation in the KcsA channel and defining its rate constants; 2) to test the consonance of the kinetic model output with experimental results.

## Methods

### Molecular Dynamics

We used the structure of the open conformation of a KcsA channel carrying the E71A mutation that prevents inactivation (PDB code 5VK6, (Cuello et al., 2017)). Previous MD results have shown that both the SF and the intracellular gate of this channel remain in an open-conductive configuration following time extensive simulations (Li et al., 2018). The channel protein was embedded in a membrane with 200 POPC lipid molecules, having a dimension of 100 × 100 Å. The system was solvated in two steps, and the distance between the maximum and minimum z coordinates and the water box edges in the *z*-axis was set to 12 Å. First, the system was solvated below the membrane plane with a water box of dimensions 102.69, 101.01, and 12.86 (x, y, and z in Å), using 11,508 TIP3 water (Jorgensen et al., 1983) molecules. Then, it was solvated above the membrane plane with a water box of dimensions 102.69, 101.01, and 12.76 (x, y, and z in Å), using 11,508 TIP3 water molecules. After the addition of the water molecules, the system presented a total net charge of 14.0 e_0_. The system was then neutralized by adding in a total of 100 Cl^-^ ions plus 86 K^+^ ions, ending up with a salt concentration of 0.4 mol/L.

The MD simulations in the present study were performed employing the NAMD molecular dynamics package (Phillips et al., 2005). The CHARMM36 force field (MacKerell et al., 1998; Best et al., 2012), with no NBFIX modification, was used in all MD simulations. An initial minimization (2,000 steps) was performed with explicit solvent using the TIP3 water model in the NpT ensemble. A distance cut-off of 12.0 Å was applied to short-range, non-bonded interactions, and 10.0 Å for the smothering functions. Long-range electrostatic interactions were treated using the particle-mesh Ewald (PME) method (Darden et al., 1993). Annealing was then performed by raising the temperature from 60 to 300 K, using a simulated temperature ramp of 0.24 ns. The pressure was maintained at 1 atm using the Nosé-Hoover Langevin piston (Martyna et al., 1994; Feller et al., 1995). A distance cut-off of 12.0 Å was applied to short-range, non-bonded interactions, and 10.0 Å for the energy switching function. Long-range electrostatic interactions were treated using the PME method. The equations of motion were integrated using the r-RESPA multiple time step scheme (Phillips et al., 2005) to update the short-range interactions every 1 step and long-range electrostatic interactions every 2 steps. The time step of integration was chosen to be 2 fs for all simulations. In this step consisting of 0.29 ns of simulation, all the backbone protein atoms and the K^+^ ions in the selectivity filter (in S0 to S4) and immediately outside were restrained. After the annealing, a 1 ns equilibration was performed in which the temperature was maintained at 300 K using Langevin dynamics. Also, during this simulation time, all the backbone protein atoms and the K^+^ ions in the selectivity filter (in S0 to S4) and immediately outside were restrained (with a force constant of 1 kcal/(mol*Å^2^)). Finally, a fully unrestrained MD simulation was performed.

*Adaptive biasing force*. The energy profiles associated with the movement of K^+^ ions along the selectivity filter were assessed using the adaptive biasing force (ABF) method which allows the calculation of the free energy variation along a coordinate of reaction (Darve et al., 2008; Hénin et al., 2010). ABF is based on the computation of the potential of mean force (PMF) along the reaction coordinate ξ, which is neutralized by the equal and opposite *biasing force* which enables the system to escape from the free energy minima, which otherwise would not allow to study the whole energy landscape. In fact, the biasing force yields a uniform transition coordinate, with only minimal residual barriers that can be easily crossed only owing to thermal fluctuations. The application of the ABF method preserves the main dynamic characteristics, including the random fluctuating force, while flattening the potential of mean force to erase free-energy barriers and, in this way, promoting the transitions between states. All this is done in an adaptive manner, without the need for prior information on the PMF (Comer et al., 2015). This kind of simulation is performed with no constraint in the coordinate reaction ξ, which implies that during the simulation the complete reaction path is discretized into small increments δξ that are explored in a continuous fashion. We started these simulations from the already equilibrated structure of KcsA mentioned earlier and proceeded with the ABFs. To allow the system to stabilize, a total of 30 ns simulation was performed, at the end of which we verified that the assessed energy profile was stable and did not change with simulation time. Furthermore, to achieve a high-resolution profile, we performed all the ABF using a δξ of 0.05 Å. During this simulation time, the backbone protein atoms of the channel transmembrane helices, sufficiently far from the selectivity filter, were restrained to a specific range of motion.

*Free energy perturbation*. To assess the binding free energy for the K^+^ ions in the sites of the SF, which is described as $\Delta G_{binding}=\Delta G_{site}-\Delta G_{bulk}$ , we performed the free energy perturbation (FEP) (Pearlman, 2002), a technique that allows to calculate the free energy variation of a system in which progressive perturbation changes occur starting from an initial state λ = 0, to get to the final state λ = 1. We can refer to this technique as a dual topology approach since both the initial and the final states are defined. As the MD progresses, the potential energy function characteristic of λ = 0 is scaled into that representative of λ = 1 by increments of δλ = 0.05. We run the simulation with a total of 35 ps for each δλ, both for the K^+^ ions in the bulk water and in the sites to get the binding free energy. The first 15 ps were excluded from the energy ensemble average calculation to allow the system to equilibrate. Soft-core potentials were used with a shifting coefficient for the van der Waals radii of 2.0 Å. Free energy differences were evaluated using the simple overlap sampling (SOS) algorithm of the ParseFEP plugin of VMD (Liu et al., 2012). Six different FEP values were averaged for each system.

### Continuum Model

Continuum models like Poisson–Boltzmann (PB) and Poisson–Nernst–Planck (PNP) equations can generally describe ion channels at equilibrium and non-equilibrium conditions, and therefore they can predict long-range behavior and stable configurations of the SF (Eisenberg, 1998; Nonner et al., 1999). However, considering ions as points without volume can deter their applicability under particular conditions, including the narrow SF of the KcsA channel where the negative charges carried by carbonyl oxygens are so strong to bring K^+^ ions to saturation levels inside it. This calls for modifications of the classical PB/PNP model to account for the steric effect of ions. To study the equilibrium situation, we consider, here, the Bikerman–PB model as one such alternative to the classical PB model. Moreover, as ion solvation energy is significant for K^+^ ions entering the narrow SF, this energy, calculated with the Born model, was included in our modeling.

**FIGURE 1:**
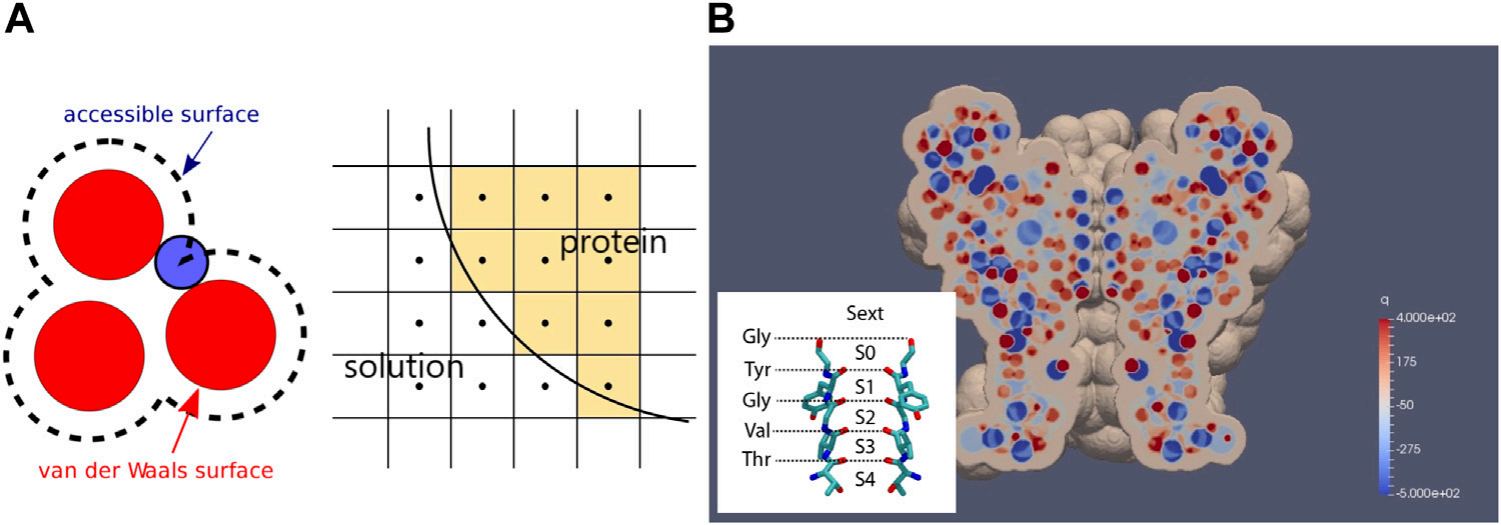
Geometry of the continuum model. **(A)** generation of protein domain by rolling ball mechanism. A mesh with its center inside an accessible surface is classified as a protein mesh. Otherwise, the mesh is classified as a solution mesh. **(B)** cross-section of KcsA with permanent charge distribution. The structure of the narrowest part of the channel, the SF, surrounded by carbonyl oxygens from the TVGYG motifs, with its binding sites, is shown in the inset. Note that unlike conventional notation, here, blue spots denote negative charges and red spots denote positive charges according to the color bar.

*Geometry.* Using the mutant E71A KcsA structure and the water molecule with a radius of 1.4 Å as the rolling ball, we generated the protein domain in our Cartesian computational mesh as illustrated in Figure 1A. A cross-section of the channel together with the distribution of its permanent charges are shown in Figure 1B. Note the narrow SF surrounded by carbonyl oxygens, with its structure shown in the inset. The membrane was further compensated to surround the channel protein in our rectangular computation domain, $\text{Ω}=\left[x_{min},x_{max}\right]\times \left[y_{min},y_{max}\right]\times \left[z_{min},z_{max}\right]$ . Therefore, the whole computational domain Ω consists of the protein/membrane domain ${\text{Ω}}_{p}$ and the electrolyte solution domain ${\text{Ω}}_{s}$ .

*Bikerman*–*PB model.* The Helmholtz free energy for the classical PB model is

F=U−T S, (1)

with the internal energy *U* and entropy *S* described as follows:

$$U=\int \left[-\frac{\varepsilon }{2}{\left|\nabla \phi \right|}^{2}+z_{p}ep\phi +z_{n}en\phi +q\phi +pW_{sol,p}+nW_{sol,n}\right]\;dV,$$ (2)

$$-TS=\int k_{B}T\left[plog\left(p\right)-p+nlog\left(n\right)-n\right]\;dV,$$ (3)

where ϕ is the electric potential; *p* and *n* are concentrations of cation and anion (K^+^ and Cl^−^ here); $z_{p}$ and $\;z_{n}$ are valencies of cation and anion; *q* is the permanent charge of protein; *e* is the elementary charge; $\varepsilon =\varepsilon_{0}\varepsilon_{r}$ is permittivity with $\varepsilon_{0}$ being the permittivity in a vacuum and $\varepsilon_{r}$ the dielectric constant (relative permittivity); $W_{sol,p},\;\;W_{sol,n}$ are the solvation energy for cation and anion, respectively; *T* is the temperature. The variation of *F* with respect to ϕ gives the Poisson equation,

$$-\nabla \cdot \left(\varepsilon \left(x\right)\nabla \phi \right)=z_{p}ep+z_{n}en+q.$$ (4)

By doing the variation of *F* with respect to *p* and *n*, we obtain the chemical potentials for cation and anion, respectively.

$$\frac{\partial F}{\partial p}=\mu_{p}=z_{p}e\phi +k_{B}Tlog\left(p\right)+W_{sol,p},$$ (5)

$$\frac{\partial F}{\partial n}=\mu_{n}=z_{n}e\phi +k_{B}Tlog\left(n\right)+W_{sol,n},$$ (6)

where solvation energy, based on the Born model, for cation and anion is

$$W_{sol,i}=\frac{z_{i}^{2}e^{2}}{8\pi \varepsilon_{0}r_{i}}\left(\frac{1}{\varepsilon_{r}(x)}-1\right),\;i=p,n,$$ (7)

with $r_{i}$ being the radius of ion *i.*

Herein, we include the steric effect to improve the classical PB model mentioned earlier. The steric effect has long been approached in modeling by modifying either internal energy or entropy in free energy (Bikerman, 1942; Borukhov et al., 1997; Horng et al., 2012). Through the entropy approach, Bikerman (Bikerman, 1942) modified classical Boltzmann distribution by adjusting bulk and local ion concentrations via excluded volume. Borukhov, Andelman, and Orland rigorously derived the same formula by adding solvent entropy through excluded volume into free energy (Borukhov et al., 1997). The Bikerman model has been a popular steric model due to its easiness of application and qualitatively good agreement with experiments. The original Bikerman model has no size distinction among ions, and all ion sizes are designated as $a^{3}$ . Many subsequent studies have extended the Bikerman model to include specific ion size via modification of the chemical potential described in (5) and (6) to the following,

$$\mu_{p}=z_{p}e\phi +k_{B}T\left[log\left(pa_{p}^{3}\right)-log\left(1-pa_{p}^{3}-na_{n}^{3}\right)\right]+W_{sol,p},$$ (8)

$$\mu_{n}=z_{n}e\phi +k_{B}T\left[log\left(na_{n}^{3}\right)-log\left(1-pa_{p}^{3}-na_{n}^{3}\right)\right]+W_{sol,n},$$ (9)

where $a_{p}^{3}$ and $a_{n}^{3}$ are volumes for cation and anion, respectively. This adjustment was rigorously derived and discussed in the review article by Horng (Horng, 2020).

At equilibrium, the chemical potential is uniform everywhere and

$$\mu_{p}=\mu_{p,b},\;\mu_{n}=\mu_{n,b},$$ (10)

with

$$\mu_{p,b}=k_{B}T\left[log\left(p_{b}a_{p}^{3}\right)-log\left(1-p_{b}a_{p}^{3}-n_{b}a_{n}^{3}\right)\right]+W_{sol,p,b},$$ (11)

$$\mu_{n,b}=k_{B}T\left[log\left(n_{b}a_{n}^{3}\right)-log\left(1-p_{b}a_{p}^{3}-n_{b}a_{n}^{3}\right)\right]+W_{sol,n,b},$$ (12)

where the subscript *b* denotes the bulk situation. Note that ϕ is set to 0 in Eqs. (11) and (12) conventionally for the bulk situation. Eqs. (8)–(12) can solve for *p* and *n*:

$$p=\frac{p_{b}e^{-\beta z_{p}e\phi }e^{-\beta \Delta W_{sol,p}}}{1+p_{b}a_{p}^{3}\left(exp\left(-\beta z_{p}e\phi \right)-1\right)+n_{b}a_{n}^{3}\left(exp\left(-\beta z_{n}e\phi \right)-1\right)},$$ (13)

$$n=\frac{n_{b}e^{-\beta z_{n}e\phi }e^{-\beta \Delta W_{sol,n}}}{1+p_{b}a_{p}^{3}\left(exp\left(-\beta z_{p}e\phi \right)-1\right)+n_{b}a_{n}^{3}\left(exp\left(-\beta z_{n}e\phi \right)-1\right)},$$ (14)

where $\beta =\frac{1}{k_{B}T},\;\;$ and

$$\Delta W_{sol,i}=\frac{z_{i}^{2}e^{2}}{8\pi \varepsilon_{0}r_{i}}\left(\frac{1}{\varepsilon_{r}(x)}-\frac{1}{\varepsilon_{r,b}}\right),\;i=p,n.$$ (15)

Readers are referred to Horng (2020) for detailed derivation. Note that with $a_{p}^{3}\to 0,\;\;a_{n}^{3}\to 0$ , Eqs. (13) and (14) reduce to classical PB distribution,

$$p=p_{b}e^{-\beta z_{p}e\phi }e^{-\beta \Delta W_{sol,p}},$$ (16)

$$n=n_{b}e^{-\beta z_{n}e\phi }e^{-\beta \Delta W_{sol,n}},$$ (17)

Eq. (4) together with Eqs. (13) and (14) form the governing equation to solve for ϕ in Ω and p,n in ${\text{Ω}}_{s}$ . Boundary conditions for ϕ at equilibrium are

$$\phi =\text{0}\;\text{at}\;{z=z}_{\min}{\text{, }z}_{\max},$$ (18)

meaning no voltage bias applied across the channel. No-flux boundary conditions are applied to four sides of the computational domain,

$$\frac{\partial \phi }{\partial n}=0\;\text{at}\;x=x_{min},\;x_{max},\;\text{and}\;y=y_{min},\;y_{max},$$ (19)

where n denotes the outward normal direction**.** The interface conditions between electrolyte and protein/membrane are

$$\left[\phi \right]=0,\;\left[\varepsilon_{r}\frac{\partial \phi }{\partial n}\right]=-\sigma ,\;\text{at}\;\text{Γ}={\text{Ω}}_{p}\cap {\text{Ω}}_{s},$$ (20)

where [⋅] denotes the jump across the interface Γ and σ is the surface density of permanent charge at the interface. Here, σ=0 due to the rolling ball scheme. For computational efficiency, Eq. (4) is augmented to be pseudo-time-dependent,

$$\frac{\partial \phi }{\partial t}=\nabla \cdot \left(\varepsilon \left(x\right)\nabla \phi \right)+z_{p}ep+z_{n}en+q.$$ (21)

We then solve for Eq. (21), instead of Eq. (4), until the steady state is reached. By the framework of the method of lines (MOL), finite volume method (FVM) is first used for the spatial discretization of Eq. (21). Then, the resultant system of ordinary differential equations (ODEs) after semi-discretization in space is integrated in time by RK4 until the steady state is reached.

As mentioned earlier, the steric effect and solvation energy difference are significant inside SF. Herein, we isolate the SF domain from the electrolyte solution domain by defining $\Omega_{\mathrm{SF}}=\Omega_{S}\cap \left\{\mathrm{z|z\in}\left[\mathrm{-4}\mathrm{.15, 11}\mathrm{.12}\right]\right\}$ . The physical and numerical parameters used in the current investigation are

1) Bulk solution concentration: ${\text{c}}_{\text{0}}=100\text{mM}$ .
2) Dielectric constant distribution: $\varepsilon_{r,bulk}=80$ at ${\text{Ω}}_{s}\backslash {\text{Ω}}_{SF}$ ; $\varepsilon_{r,p}=2$ at ${\text{Ω}}_{p}$ ; and $\varepsilon_{r,SF}=1.5$ at ${\text{Ω}}_{SF}$ . A sharp linear transition from bulk $\varepsilon_{r,bulk}$ to $\varepsilon_{r,SF}$ is designated at both edges of ${\text{Ω}}_{SF}$ as shown in Figure 3A. Debye length based on $\varepsilon_{r,SF}$ is $\lambda_{D,SF}=\sqrt{\frac{\varepsilon_{0}\varepsilon_{r,SF}k_{B}T}{c_{0}e^{2}}}=1.89Å$ .
3) Ion diameter: $a_{K^{+}}=2.76Å,\;\;\;a_{Cl^{-}}=3.62Å,\;\;\;a_{water}=2.8Å$ .
4) Bulk diffusion coefficients: $D_{K}=1.957\times 10^{-9}\;m^{2}/s,\;\;\;D_{Cl}=2.032\times 10^{-9}\;m^{2}/s$ .
5) Grid size: Δx=Δy=Δz=0.2Å .
6) Computational domain: $\text{Ω}=\left[x_{min},x_{max}\right]\times \left[y_{min},y_{max}\right]\times \left[z_{min},z_{max}\right]$ with $x_{min}=-30,\;x_{max}=30,y_{min}=-30,y_{max}=30,z_{min}=-38,z_{max}=35.6\;$ in  Å .

Note that $\lambda_{D,SF}$ is generally larger than the width of SF, which means that the electric double layers (EDLs) in the SF will overlap. Dielectric constant inside SF, $\varepsilon_{r,SF}$ , is practically hard to estimate by MD or be measured experimentally. So, here, we treated it as a model parameter. In the beginning, we only knew water inside SF should be far less than in the bulk, and therefore the value should be far less than 80. We have conducted simulations with $\varepsilon_{r,SF}$ being set to 10, 8, 6, 4, 2, and 1.5 and discovered the less occupation of S1 and S4 by K^+^ ions as $\varepsilon_{r,SF}$ decreases mainly due to the increasing solvation energy barrier based on the Born model, Eq. 15. The value 1.5 gives a complete absence of K^+^ ions at S1 and S4 which agrees best with our MD equilibrium result shown in a later section.

### Solution of the Kinetic Model

The solution of the kinetic model was obtained by assuming that the system is at steady state, meaning that the rate of formation of each state is equal to the rate of disappearance, giving a zero rate of change. More specifically, considering a kinetic model having N different states, under steady-state conditions, we can write a system of N equations of the type

$$\underset{j}{\sum }k_{ji}\;n_{j}-\underset{j}{\sum }k_{ij}\;n_{i}=0\;\text{for}\;\text{i}=1.........\text{N,}$$ (22)

where *k*_*ji*_ is the rate constant going from state *j* to state *i*, and *n*_*j*_ is the fractional occupancy of state *j*, with *j* that varies over all the states connected with state *i.* Using this system of equations and considering the additional constraint that the sum of fractional occupancies is unity, $\overset{N}{\underset{i=1}{\sum }}n_{i}=1$ , we can find the unknown fractional occupancies of the various states that can then be used to assess the ion current as follows:

$$current=z_{K}\;e\;\underset{i,\;j}{\sum }\left(k_{ij}\;n_{i}-k_{ji}\;n_{j}\right),$$ (23)

where $z_{K}$ is the valence of a K^+^ ion (+1), and *i* and *j* vary for all possible transitions *i*→*j* representing either the entry of a K^+^ ion from the intracellular bath or its exit to the extracellular bath.

In our specific case, the kinetic scheme shown in Figure 4 gives rise to the following system of equations:

$$k_{a3}\;{\left[K^{+}\right]}_{ex}\;n_{4}+\;k_{b}\left(V\right)\;n_{3}+\;k_{d1}\;n_{2}-\;n_{1}\;\left(k_{a1}{\left[K^{+}\right]}_{in}+\;k_{f}\left(V\right)+k_{d3}\right)=0,$$ (24)

$$k_{a1}\;{\left[K^{+}\right]}_{in}\;n_{1}+\;k_{a2}{\left[K^{+}\right]}_{ex}n_{3}-\;n_{2}\;\left(k_{d1}+\;k_{d2}\right)=0$$

$$k_{a4}\;{\left[K^{+}\right]}_{in}\;n_{4}+\;k_{f}\left(V\right)\;n_{1}+\;k_{d2}\;n_{2}-\;n_{3}\;\left(k_{a2}{\left[K^{+}\right]}_{ex}+\;k_{b}\left(V\right)+k_{d4}\right)=0$$

$$k_{d3}\;n_{1}+\;k_{d4}\;n_{3}-\;n_{4}\;\left(k_{a3}{\left[K^{+}\right]}_{ex}+\;k_{a4}{\left[K^{+}\right]}_{in}\right)=0$$

where ${\left[K^{+}\right]}_{ex}$ and ${\left[K^{+}\right]}_{in}$ represent the extracellular and intracellular K^+^ concentrations and *n*_*i*_ represents the fractional occupancy of state *i* and $n_{1}+\;n_{2}+\;n_{3}+n_{4}=1$ . The motivation of using the four-states scheme shown in Figure 4 to describe the permeation pathway and construct the kinetic model accordingly will be explained later. The above linear system of equations was solved at varying voltages and K^+^ intracellular and extracellular concentrations, and the *n_i_* were used to assess the current as follows:

$$current=z_{K}\;e\;\left(k_{a1}{\left[K^{+}\right]}_{in}n_{1}+k_{a4}\;{\left[K^{+}\right]}_{in}\;n_{4}-k_{d1}\;n_{2}-k_{d4}\;n_{3}\right),$$ (25)

or equivalently

$$current=z_{K}\;e\;\left(k_{d2}\;n_{2}+\;k_{d3}\;n_{1}-k_{a2}\;{\left[K^{+}\right]}_{ex}n_{3}-k_{a3}\;{\left[K^{+}\right]}_{ex}\;n_{4}\right),$$ (26)

As with any cyclic kinetic model, the so-called microscopic reversibility needs to be respected at the equilibrium (zero voltage), that is, the product of the kinetic rate constants in the clockwise direction should equal the product of the rate constants in the counterclockwise direction. In our case, we have three cycles, thus the microscopic reversibility should read:

$${\text{K}}_{\text{a}1}{\left[{\text{K}}^{+}\right]}_{\text{in}}{\text{k}}_{\text{d}2}{\text{k}}_{\text{b}}\left(\text{0}\right){=\text{K}}_{\text{d}1}{\text{k}}_{\text{f}}\left(0\right){\text{k}}_{\text{a}2}\;{\left[K^{+}\right]}_{ex},$$ (27)

$${\text{K}}_{\text{d}3}{\text{k}}_{\text{a}4}{\left[{\text{K}}^{+}\right]}_{\text{in}}{\text{ k}}_{\text{b}}\left(\text{0}\right){=\text{K}}_{\text{a}3}\;{\left[{\text{K}}^{+}\right]}_{\text{ex}}{\text{k}}_{\text{f}}\left(\text{0}\right){\text{k}}_{\text{d}4}$$

$$K_{a1}{\left[K^{+}\right]}_{in}k_{d2}k_{d4}K_{a3}\;{\left[K^{+}\right]}_{ex}={\text{K}}_{\text{d}1}{\text{K}}_{\text{d}3}{\text{k}}_{\text{a}4}{\left[{\text{K}}^{+}\right]}_{\text{in}}k_{a2}\;{\left[K^{+}\right]}_{ex}\;$$

*Multi-scale approach for predicting IV relationships.* To predict the IV relationships for the KcsA channel, we considered the association/dissociation model shown in Figure 4, with the rate constants assessed from MD data. More specifically, we used the MD ABF method to assess the energy profiles associated with the various transitions present in the kinetic scheme and to estimate the diffusion rate constants outside and inside the selectivity filter of the channel. In order to take into account the effect of the transmembrane voltage on the voltage dependent rate constants of the model, *k*_*b*_ and *k*_*f*_, an electrostatic energy corresponding to a linear voltage drop through the SF was added to the energy profile. Once these parameters are known, the kinetic rate constants can be estimated using Eqs. (28) and (29), to be described, and the currents under various voltages and K^+^ concentrations using Eqs. (25) and (26).

## Results

### Molecular Dynamics of K^+^ Permeation in KcsA Channel

**FIGURE 2:**
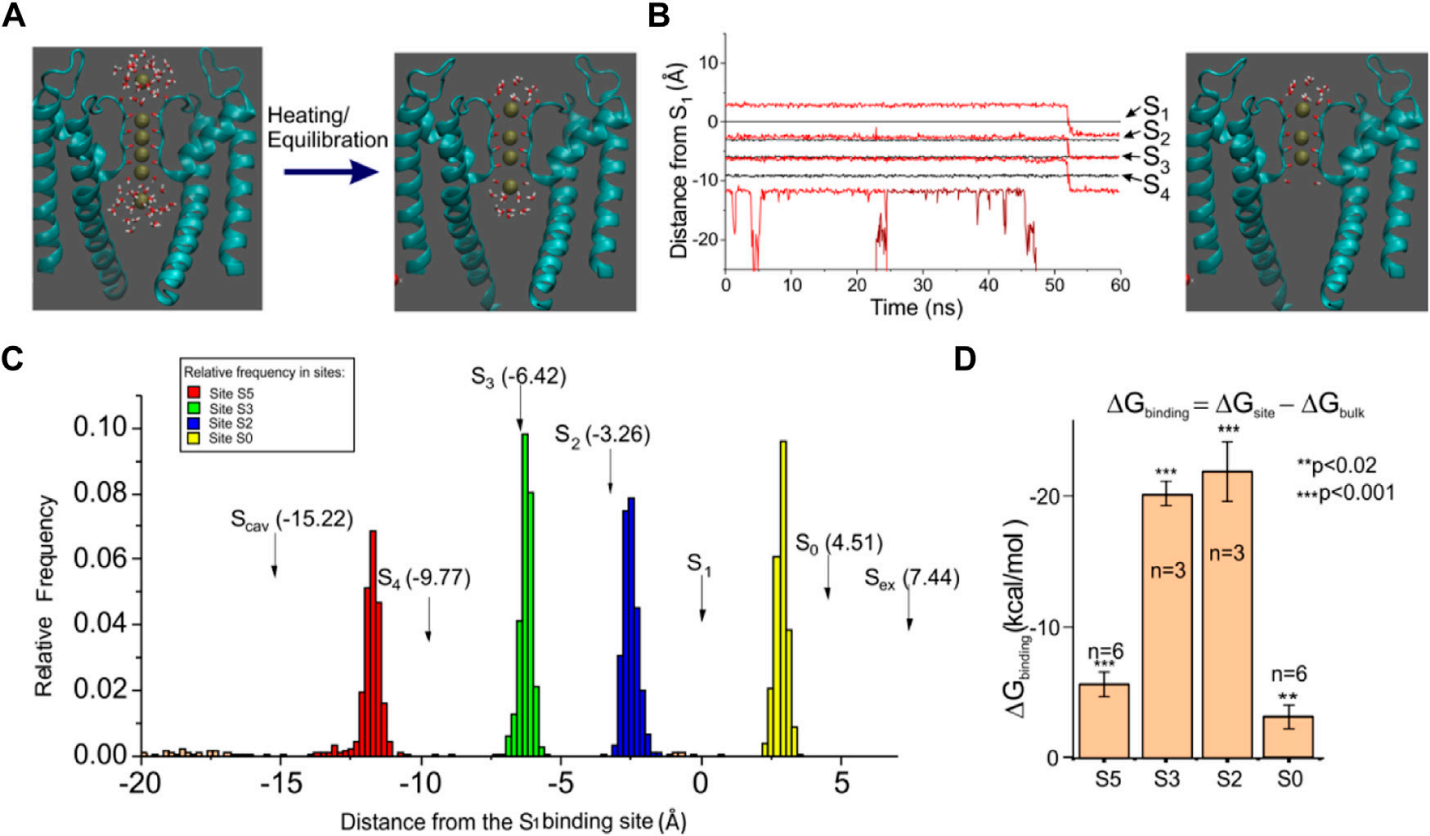
Molecular dynamics of the KcsA channel. **(A)** *left:* KcsA structure and initial K^+^ ion configuration used in our MD simulations. K^+^ ions were placed in sites S1–S4 inside the selectivity filter, at the cavity, and in the site S_ex_ found in X-ray crystallography. *Right:* typical ion configuration found after heating and equilibration. **(B)** *left:* plot of the K^+^ ion position as a function of time for one typical MD simulation. The black lines represent the position of the four SF crystallographic sites (assessed as the center of mass of the eight carbonyl oxygens) while the red lines represent the position of the K^+^ ions. *Right:* SF configuration found at 50 ns, with only three K^+^ ions bound to the SF. **(C)** amplitude histogram of the K^+^ ion position assessed from a 400 ns long MD simulation, showing four preferential positions. The arrows indicate the K^+^ binding sites found in the high-resolution structure. **(D)** binding free energy assessed using the free energy perturbation technique for the four K^+^ binding sites found to be typically occupied in our simulations. The plot reports the difference between the free energy variation obtained following the annihilation of a K^+^ ion in water (ΔG_bulk_) and a K^+^ ion sitting in a binding site (ΔG_site_). In the initial configuration, shown on the left of panel A, ions were constrained within +/- 1 Å from the binding site using a collective variable approach of NAMD. Since the application of a constraint adds an entropy term to the free energy variation (Wang et al., 2006), the same constriction was also applied to the bath K^+^ ion, in order to have the same effect on all free energy values presented.

We performed MD simulations starting from the newly determined crystallographic configuration of the selectivity filter of KcsA, with four K^+^ ions sitting in the four SF binding sites, very close to each other, plus a K^+^ ion in the cavity and another one in the external vestibule (Figure 2A, left). Since this configuration was obtained from crystallized channels held at a very low temperature, we first verified if it was also present as a relatively stable configuration at more physiological temperatures, which are easily reached by heating the crystal from 60 to 300 K and letting it equilibrate for about 400 ns. We performed this simulation ten times, each time randomly re-initializing the atoms’ velocities, and every time, at the end of the equilibration, we observed a K^+^ ion configuration invariably characterized by two K^+^ ions at the two most internal binding sites of the selectivity filter (S2 and S3) (Figure 2A, right). The S1 and S4 positions were instead empty or rarely occupied by water (water occupancy was 25.2 ± 7.3% for S1 and 11.5 ± 1.4% for S4).

Two other K^+^ ions were, respectively, found at the intracellular and extracellular entry of the selectivity filter. These K^+^ ions were instead only partially hydrated, as they also interacted with the protein residues. More specifically, the K^+^ at the external site interacted with the carbonyl oxygens of tyrosine 78, located at the extracellular entry of the selectivity filter, while the K^+^ at the intracellular entrance interacted with the hydroxyl oxygens of threonine 75. As can be seen from the frequency plot of Figure 2C, neither of the two sites exactly matches the corresponding binding sites found in the crystal (Scav and S0, indicated with arrows in the plot), especially the internal site which was clearly distinct from Scav. The position where we found the internal K^+^ site corresponds very closely to the S5 site found by Jensen et al. (2010) on Kv1.2, so we will refer to it as site S5. As for the external site that was found less distant from the crystal site S0, we kept this name. In any case, these simulations consistently showed the presence of the stable 0/2/3/5 configuration in the SF at the end of equilibration (cf. Figure 2B).

We also assessed the binding free energy of the four identified K^+^ binding sites, using free energy perturbation (a technique that essentially consists in slowly annihilating the K^+^ ion interactions with the environment and assessing the free energy change observed during the process). If the free energy change is significantly higher than for the annihilation of a K^+^ ion in water, then we can arguably talk about a K^+^ binding site. The bar plot in Figure 2D shows that the free energy increase for K^+^ annihilation in all the four binding sites is systematically higher than in the bath, meaning that these are all true binding sites for K^+^. Notice, however, the considerably smaller binding free energy for both external sites S0 and S5, compared to the internal sites S2 and S3, which is arguably the basis of the high conduction rates in this channel. In several simulations, we were in fact able to observe the unbinding of K^+^ from either the S0 or S5 position (Figure 2B, K^+^ unbinding from S5, occurring at 45 ns; lowest red trace), followed by a concerted motion of the three remaining ions as a single file along the selectivity filter (Figure 2B, concerted transition from 0/2/3 configuration to 2/3/5 configuration).

### The Bikerman–PB Continuum Model of KcsA Channel

**FIGURE 3:**
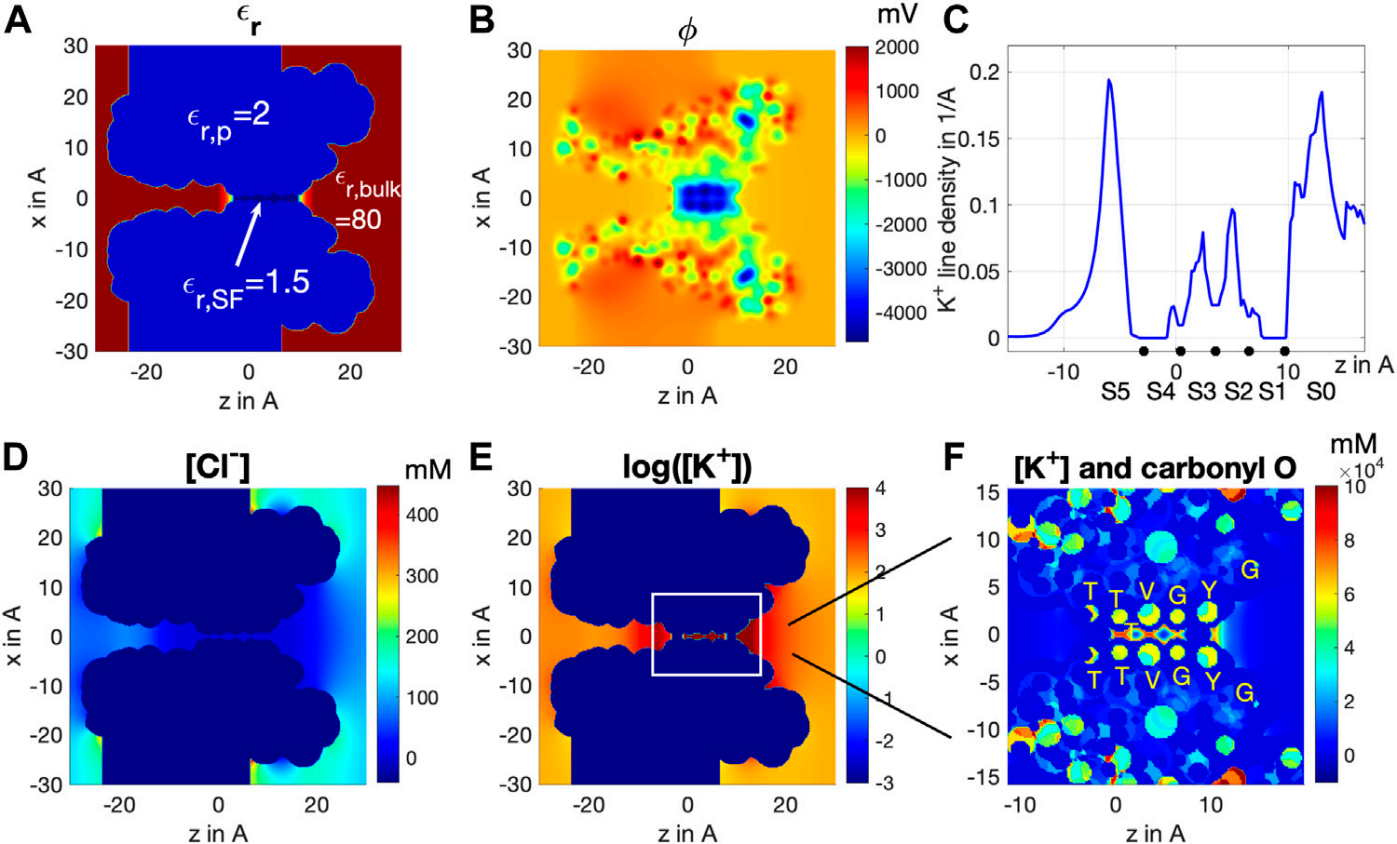
Cross-section distribution of **(A)** dielectric constant $\varepsilon_{r}$ , **(B)** electric potential ϕ , **(C)** line density distribution of K^+^ ions along the central axis, **(D)** $\left[Cl^{-}\right]$ and **(E)** $log_{10}\left[K^{+}\right]$ , at equilibrium ( V=0 ). **(F)** close-up of cross-section distribution of $\left[K^{+}\right]$ and −q around SF.

To verify if the stable 0/2/3/5 configuration, indicated by MD simulations, is a consistent occurrence at steady state, we applied the Bikerman–PB continuum model to the same channel structure used in MD simulations. PB/PNP-type continuum models can generally describe ion channels in equilibrium and non-equilibrium conditions, and therefore could be a useful integrative approach to MD, especially in predicting long-range behavior and stable configurations of the SF (Eisenberg, 1998; Nonner et al., 1999). The computation results based on the Bikerman–PB model for equilibrium situation, V=0 and symmetric K^+^, are shown in Figure 3. Figure 3A illustrates the channel’s cross-sectional view with the dielectric constant values assigned to the significant locations of the channel, namely ε_r,P_ = 2.0 in the protein matrix ( ${\text{Ω}}_{p}$ ), ε_r,SF_ = 1.5 in the SF ( ${\text{Ω}}_{SF}$ ), and ε_r,bulk_ = 80 for the channel vestibules and bulk ( ${\text{Ω}}_{V}/{\text{Ω}}_{bulk}$ ). Figure 3B shows the distribution of the electric potential ϕ , where we can observe a very negative electric potential distribution along the SF. As a consequence, K^+^ is strongly accumulated and Cl^−^ heavily depleted inside the SF and the entrances (Figures 3D, E). There are, however, two ion depletion spots at the SF, where no ions are virtually ever found (Figure 3E). These two spots are located at sites S1 and S4 of SF, as better illustrated in Figure 3F, where [K^+^] and -q (minus of permanent charges) are plotted together to show how carbonyl oxygens attract K^+^ ions, especially at their ridge where saturation peaks with [K^+^] ≈ 80M are formed. Note that this extremely high saturation concentration happening at ridges (better presented in panel A of Supplementary Figure S1) actually comes from the Bikerman model, Eqs. (8) and (9), where $log\left(1-pa_{p}^{3}-na_{n}^{3}\right)\approx \text{log}\left(0^{+}\right)$ (to be more specific $log\left(1-pa_{p}^{3}-na_{n}^{3}\right)\approx \log\left(10^{-5}\right)\approx -11.5$ according to our simulation data) with $n\approx 0^{+},\;p\approx 1/a_{p}^{3}$ when K^+^ ion saturating and Cl^−^ ion totally excluded from SF. With $a_{K^{+}}=2.76Å$ , the saturation concentration is $\left[{\text{K}}^{+}\right]\approx {1\text{/a}}_{{\text{K}}^{+}}^{3}\approx 80\text{M}$

Yet the K^+^ distribution along the central axis shown in Figure 3F cannot fully reveal the effective residence of K^+^ ions in the SF. For this reason, we integrated [K^+^] over the xy-plane cross-sectional area of the SF to obtain the line density of K^+^ ions as a real measure of the K^+^ spatial distribution along the SF, as shown in Figure 3C. Notably, we find peaks of K^+^ residence at sites S0, S2, S3, and S5, but basically none at sites S1 and S4. The fact that S0 and S5 display much larger peaks than S2 and S3 is mainly due to K^+^ ions having much more room at vestibule sites S0 and S5 than S2 and S3 do inside the SF, rather than a real difference in the concentration of the K^+^ ion at these sites, as indicated in Figure 3F. The slightly higher line density distribution at S2 than at S3 agrees with the observation from MD that S2 is a more stable binding site than S3 (Figure 2D) (Noskov et al., 2004). These results fully agree with MD simulations reported in Figure 2, showing the presence of a stable 0/2/3/5 configuration. Significant physics stays behind the stability of the 0/2/3/5 configuration, which is illustrated by comparing the results of the Bikerman–PB model with the classical PB model. Readers are referred to supplementary information in this article for further details.

### The Association/Dissociation (A/D) Permeation Kinetic Model in KcsA Channel

**FIGURE 4:**
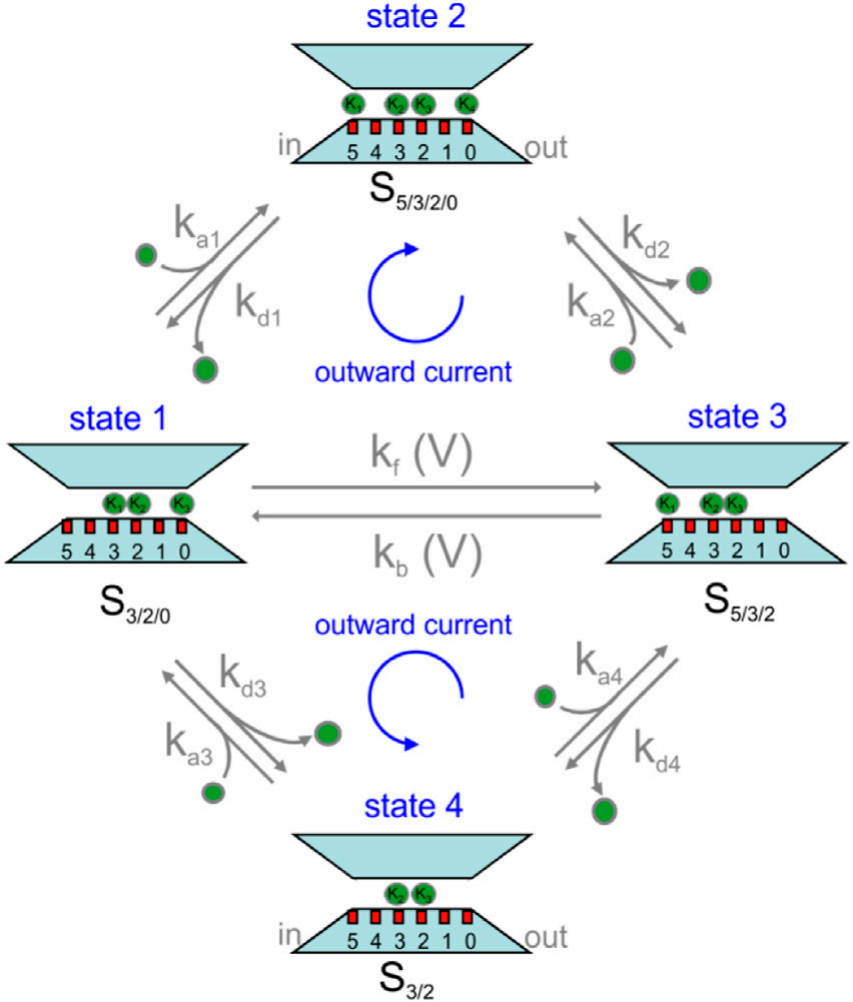
Permeation kinetic model for KcsA channel proposed in this article. See text for description.

MD and continuum modeling results shown above inspired us the A/D permeation mechanism shown in Figure 4. As we have seen, the channel shows an energetically stable configuration with sites S5, S3, S2, and S0 occupied by K^+^ ions and sites S4 and S1 empty (state 2 in Figure 4, also cf. first 45 ns simulation of Figure 2B). From this 4-K^+^ ion configuration, the channel can easily exchange with the bath either the K^+^ ion in S5 or in S0, as indicated by the binding energetics of Figure 2D. To verify if the stable 0/2/3/5 configuration, indicated by MD simulations, is a consistent occurrence at steady state, we applied the Bikerman–PB continuum. We also considered the occurrence that both K^+^ ions in S0 and S5 unbind in rapid succession, and in any case, before either ion in S2 or S3 moved, leaving the selectivity filter in the 2-K^+^ configuration (state 4) that we have observed in our MD simulations (data not shown, see also the supplementary movie in (Köpfer et al., 2014)).

From the above considerations, K^+^ permeation can be viewed as a K^+^ ion binding to either one of the two external sites, S5 or S0, in states 1 or 3, and another K^+^ ion being released (unbinding) from the opposite site (upper part of the scheme). In fact, the two events may also occur in the reverse order, with first the unbinding of a K^+^ ion and then the binding of another K^+^ at the opposite site (lower part of the scheme). In order for the system to give a continuous K^+^ flux, we finally need to picture how the two 3-K^+^ ion configurations, state 1 and state 3, can interconvert into one another through a single file movement, considering that in our model this inter-conversion must be voltage dependent. In fact, it is the only voltage-dependent process, given that the voltage applied across the membrane drops mostly inside the selectivity filter (Catacuzzeno et al., 2020), thus leaving the binding and unbinding of K^+^ to and from sites S0 and S5 essentially outside the electric field. We are aware that the states of our permeation model shown in Figure 4 are mainly derived from MD simulations under equilibrium conditions. It is, however, reassuring that a recent study performed on the same E71A mutant KcsA channel, under non-equilibrium conditions, has come up with essentially the same model (Domene et al., 2021).

To verify whether the permeation mechanism considered can predict the experimental current-voltage relationships, we estimated the model rate constants through MD simulations. For rate constants not involving the binding of K^+^ ions, namely the inter-conversion rates between states 1 and 3 and all the dissociation constants, *k*_*d*_s, we used the mean first-passage time (MFPT) theory, originating from the Langevin’s diffusion model (Szabo, 1979; Cooper, Gates, and Eisenberg, 1988a; Cooper, Gates, and Eisenberg, 1988b; Schulten et al., 1981). According to this theory, a rate constant *k* can be estimated as the inverse of the mean first-passage time, that is, the time needed to go from the initial state *i* to the final state *f* of the transition.

$$k=\;{\left[\tau_{f}+\tau_{b}\;\frac{Z_{f}}{Z_{b}}\right]}^{-1},$$ (28)

$$Z_{f/b}=\overset{b}{\underset{i/f}{\int }}e^{-\frac{U\left(y\right)}{kT}}dy$$

$$\tau_{f/b}=\frac{1}{D}\overset{b}{\underset{i/f}{\int }}dx\;\overset{b}{\underset{x}{\int }}e^{-\frac{U\left(y\right)-U\left(x\right)}{kT}}dy$$

where *U(x)* is the energy profile associated with the process, *D* is the diffusion constant, and *k* and *T* have their usual meanings. *i*, *b*, and *f* represent respectively the initial position, the point of maximal energy, and the final position along the reaction coordinate.

As for the K^+^ association rate constants, *k*_*a*_ *s*, we assumed that they are diffusion-limited and assessed their values from the following equation derived from the Smoluchowski (Debye, 1942):

$$k_{a}=2\;\pi \;D\;{\left[\overset{\infty }{\underset{r_{c}}{\int }}\frac{\exp(U\left(r\right)/kT)}{r^{2}}\;dr\right]}^{-1},$$ (29)

where *r*_*c*_ is the radius of the hemisphere through which K^+^ can enter the pore, here considered to be 1.5 Å and *D* is the bulk K^+^ diffusion coefficient. Notice that Eq. (29) is valid only under the assumption that K^+^ ions can freely diffuse to the binding site. While this is probably correct for the binding site S0, the binding to site S5 likely involves K^+^ diffusion through the more restricted region of the intracellular hydrophobic pore; thus, our estimate of *k*_*a1*_ and *k*_*a4*_ and the resulting estimated current would likely represent an upper limit.

As can be seen from Eqs. (28) and (29), the association rate constant depends obviously on the energy profile encountered during the process but also on the diffusion constant of K^+^ ions approaching the site involved in the transition. We estimated the K^+^ diffusion coefficient, both in the bath and the selectivity filter, from MD simulations, as the mean squared fluctuation of the K^+^ ion position along the *z*-direction (Bernèche and Roux, 2001; Bullerjahn et al., 2020). It may be noticed that the value assessed for K^+^ ions in the bath (0.235 ± 0.021 A^2^/ps) is slightly higher (yet within 20%) than the experimental value of 0.196 A^2^/ps. Inside the selectivity filter, we obtained, as expected, a much lower value for the diffusion coefficient, namely 0.040 ± 0.005 A^2^/ps, in substantial accordance with previous measurements (Allen et al., 2000).

**FIGURE 5:**
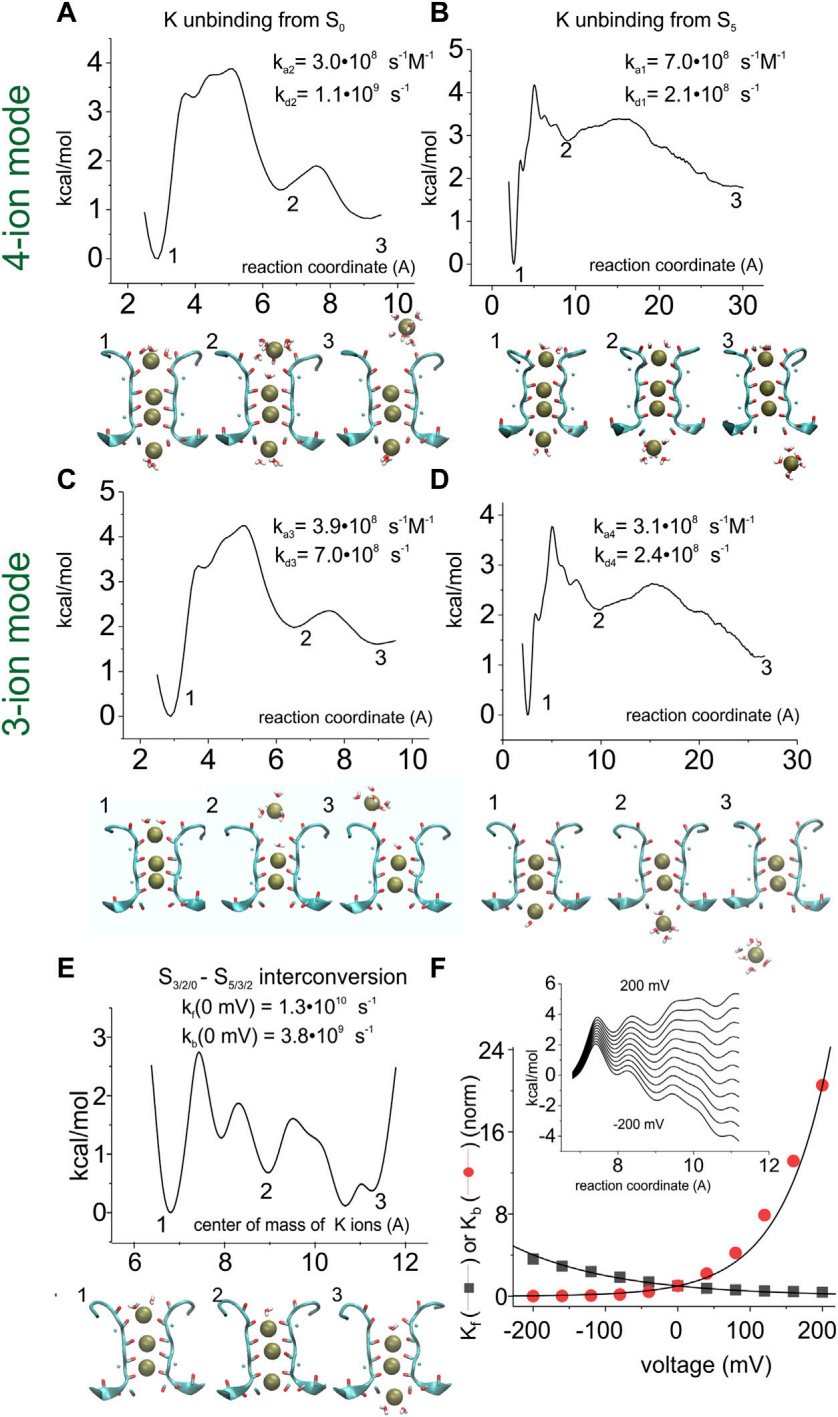
Assessment of the energy profiles and kinetic rate constants for the K^+^ permeation kinetic model. Energy profiles were assessed by using the adaptive biasing force (ABF) 1D method for the K^+^ unbinding from S0 into the extracellular solution (A when starting from a four-ion configuration, and C when starting from a three-ion configuration of the SF), from S5 into the intracellular solution (BD), and for the interconversion of the three K^+^ ions inside the SF **(E)**. In **(A)** and **(C)**, the reaction coordinate is the distance between the most extracellular K^+^ ion and the center of mass of the carbonyl oxygens forming site S1. In **(B)** and **(D)**, the reaction coordinate is the distance between the most intracellular K^+^ ion and the center of mass of the carbonyl oxygens forming site S4. In **(E)**, the reaction coordinate is the distance between the center of mass of the three K^+^ ions and the center of mass of the carbonyl oxygens of residue 79. **(F)** *inset*: energy profile for the 3-K^+^ ion configuration of the selectivity filter, plotted as in panel E but with the addition of linear electrostatic energy of variable amount (corresponding to potential differences from -200 to +200 mV). Compared to panel E, only the part of the energy profile going from configuration S3/S2/S0 (energy well #1) to configuration S5/S3/S2 (energy well #3) is reported. Main plot: forward and backward rate constants (K_f_ and K_b_ in the scheme of Figure 4, respectively) as a function of voltage, assessed from the energy profiles shown in the inset, using Eq. (28). The numbers associated with certain wells in the energy profiles define the configuration shown below the graphs.

We then estimated the energy profile associated with each of the transitions present in the reaction scheme. To this end, we used the adaptive biasing force (ABF) method, where the energy profile is adaptively assessed as the force needed to obtain a uniform distribution of the system along the reaction coordinate. Starting from the uneven distribution of K^+^ ions due to their accumulation at the energy wells of the actual energy profile, additional energy is applied and changed adaptively until the K^+^ distribution of the system is homogeneous along the reaction coordinate, meaning that the biasing energy exactly compensates for the original energy profile. The inverse of the biasing energy is then taken as the energy profile characterizing the process. Figure 5 shows the application of this method to assess the energy profiles associated with the various reaction steps present in the model considered. In the case of the interconversion between the 3-K^+^ ion configurations (Figure 5C), we performed a mono-dimensional ABF in which the reaction coordinate is the center of mass of the three K^+^ ions.

Since we assumed the 3-K^+^ interconversion process to be voltage dependent, the assessment of the rate constants was performed both with the original energy profile and after adding various electric potential differences linearly dropping along the entire profile (Figure 5F inset). The plot in Figure 5F reports the estimated rate constants as a function of the applied potential.

As with any cyclic reaction, the model considered must respect the principle of microscopic reversibility, imposing that any molecular process and its reverse occur at equal rates, at equilibrium. Notably, the estimated rate constants were quite close to this condition, so we had to modify them only slightly (less than 25%, manually adjusted) to get perfect microscopic reversibility (Table 1).

**TABLE 1:** Rate constants evaluated from MD and adjusted by microscopic reversibility.

Rate constant	Value derived from MD (x 10^8^)	Value adjusted for microscopic reversibility (x 10^8^)
k_a1_	7.0 s^−1^M^−1^	5.0 s^−1^M^−1^
k_d1_	2.1 s^−1^	2.5 s^−1^
k_a2_	3.0 s^−1^M^−1^	4.0 s^−1^M^−1^
k_d2_	11.0 s^−1^	10.0 s^−1^
k_a3_	3.9 s^−1^M^−1^	4.0 s^−1^M^−1^
k_d3_	7.0 s^−1^	10.0 s^−1^
k_a4_	3.1 s^−1^M^−1^	4.0 s^−1^M^−1^
k_d4_	2.4 s^−1^	2.0 s^−1^
k_f_(0)	130.0 s^−1^	150.0 s^−1^
k_b_(0)	38.0 s^−1^	30.0 s^−1^

To assess the effects of the diffusional restriction imposed by the intracellular vestibule, we also used the following Smoluchowski version of the association rate constant, where the accessibility to the S5 site is considered through a cylindrical hole of radius $r_{v}$ instead of a hemispherical surface:

$$k_{a}=\pi \;D\;r_{v}^{2}{\left[\overset{l_{v}}{\underset{r_{c}}{\int }}\exp\left(U\left(r\right)/kT\right)\;dr\right]}^{-1},$$ (30)

Here, $l_{v}$ represents the length of the intracellular vestibule, approximately 20 Å, and $r_{c}$ , as before, represents the distance of the adsorbing surface from the binding site, taken as the position of the maximum value along the energy profile. Using this equation, we found that the K^+^ association binding constant $k_{a1}$ , that has a value of 7 × 10^8^ s^−1^ M^−1^ assuming a hemispherical absorbing surface, changes to 4.9 × 10^8^ s^−1^ M^−1^ with an $r_{v}$ of 13.5 Å (chosen to get a Cα- Cα distance of Thr112 of 32 Å, as in the 3f5w structure) and to 1.9 × 10^8^ s^−1^M^−1^ using an $r_{v}$ of 8.5 Å (chosen to get a Cα-Cα distance of Thr112 of 22 Å, as in the 5vk6 structure). Since currents at very high voltages and relatively high concentrations linearly depend on $k_{a1}$ based on Eq. 25, these results suggest that the current predicted would depend on the KcsA structure used.

**FIGURE 6:**
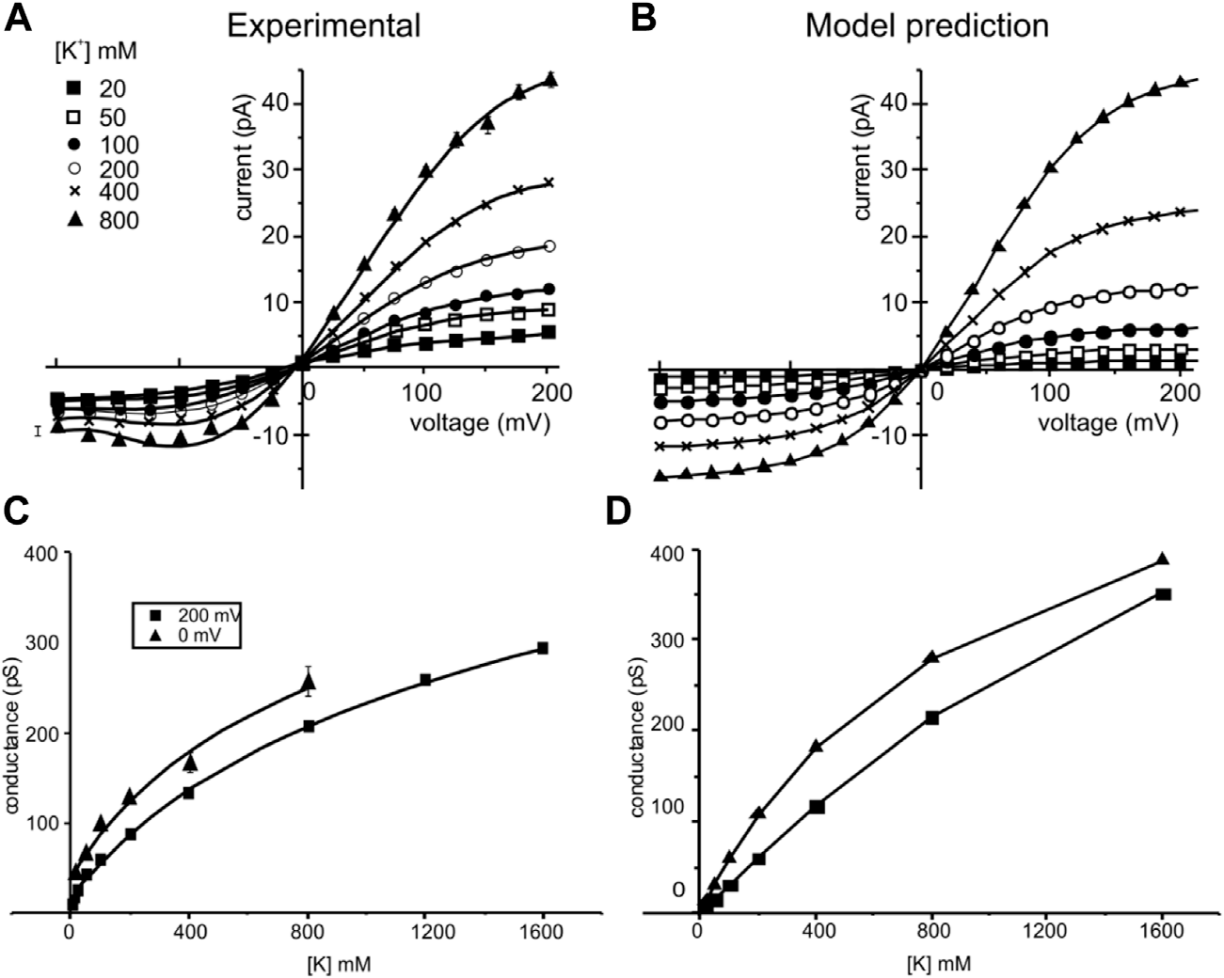
Experimental and simulated current-voltage (IV) and conductance-concentration plots. *Left*: experimental IV relationships (top) of the KcsA channels taken at different symmetrical K^+^ concentrations (indicated) and conductance-concentration relationships (bottom) at two membrane potentials. Data are from LeMasurier et al. (2001). *Right:* IV and conductance-concentration relationships predicted by our model using the kinetic scheme of Figure 4 and rate constants adjusted for microscopic reversibility in Table 1. The conditions used in our modeling were the same as those used to obtain the experimental results.

Figure 6 top shows the predicted IV relationships at different K^+^ concentrations and compares them with those observed experimentally. Notably, all the major features of the experimental IVs, namely the amount of current carried, the rectification (less current at negative than positive voltages), the sub-linearity (saturation at highly positive and negative voltages), and the saturation of the chord conductance at high concentrations, appear quite well reproduced. Slightly lower predicted currents than those observed experimentally are instead found at relatively low K^+^ concentrations, especially at negative voltages. We think that this may originate from surface charge effects that would tend to increase the K^+^ concentration close to the channel, especially when the ionic strength is low. The bottom panels compare the conductance-concentration relationship derived at two potentials from our model with those found experimentally. Matching between experimental and modeling data is objectively good.

## Discussion

Upon heating and equilibrating a K^+^-filled SF, as observed in X-ray crystallography, we found a stable SF configuration consisting of two K^+^ ions bound to the crystallographic binding sites S2 and S3, and two additional K^+^ ions weakly bound right at the entrances of the selectivity filter, at sites not apparent in the crystal, sites S0 and S5. K^+^ ions sitting in these two sites appear only partially hydrated, as they are in part stabilized by the carbonyl oxygens of tyrosine 78 and the hydroxyl oxygens of threonine 75, respectively. The Bikerman–PB continuum model, shown to correctly predict this stable 0/2/3/5 K^+^ ion distribution, confirmed the MD-derived configuration and provided clues on the physicochemical determinants behind it.

The configurations found suggested an association-dissociation kinetic model of permeation that could reproduce the experimentally observed conductance saturation and current-voltage sub-linearity, thus showing that an A/D model of permeation could well apply to the KcsA channel. Interestingly, our proposed mechanism is very similar to that found by Domene et al. (2021) where they analyze relatively long (microseconds) MD simulations from the E71A mutant KcsA channel in terms of transition probability matrix. However, in that study, the authors attempted neither to assess the kinetic rate constants related to various SF configurations nor to verify the prediction of the experimental electrophysiological data.

Notice that the experimental data used for comparison in Figure 6 is actually for WT KcsA (Lemasurier et al., 2001), whereas our multi-scale predictions are carried out using the E71A mutant. This is because there are only a few current-voltage measurements available in the literature for E71A (Hirano et al., 2010; Rotem et al., 2010; McCoy et al., 2014), and none of them has documented current-voltage curves under a wide range of bulk concentrations as carried out by LeMasurier et al. (Lemasurier et al., 2001). WT and E71A might differ in some aspects of the permeation. Generally speaking, the difference in outward current is small between WT and E71A but larger in the inward current (Rotem et al., 2010). It has been explained that E71A mutation reduces the outward rectification by increasing inward current at strong negative voltages, suggesting that the “flipped Asp80” creates a ring of extracellular charges and increases inward current with no effects on the outward current (Rotem et al., 2010). Our results presented in Figure 6 are in accordance with this observation, though Asp80 remained in the non-flipped position, as in WT, in our MD simulation (Supplementary Figure S2). Thus, the subtle current deviation of E71A from WT at strong negative voltages may indeed reflect their minor structural differences around the selective filter. However, it is not clear if the difference can be fully explained by the Asp80 side-chain orientation.

To identify a realistic mechanism of K^+^ permeation through KcsA channels, in this study we used a multi-scale approach, whereby the information obtained from MD and from continuum models at equilibrium is used to postulate a plausible kinetic model of permeation, in terms of both the number, types, and connections between the stable configurations considered and the quantitative values of the rate constants connecting them. Once this step is completed, the kinetic model can be easily verified for its predictive capability of the permeation properties experimentally available. The approach that we used here was necessary because the assessment of the single-channel current by MD typically requires a few weeks on a common workstation, a time that needs to be scaled up correspondingly because these assessments have to be repeated at several membrane potentials to construct more informative current-voltage relationships. This high computational cost strongly limits the range of possible analyses and imposes the use of non-physiological simulation conditions in terms of membrane potentials and ion concentrations. The second shortcoming of an approach fully based on MD simulations is the overwhelming number of details that the resulting trajectories provide (i.e., the positions and the velocities of all the atoms over time) most of which are not relevant for the conduction and selectivity mechanisms. On the contrary, they might be harmful as they could hinder the identification of the effectively relevant features for the process under investigation. The multi-scale approach proposed in this study tries to overcome these two current shortcomings of MD simulations on computational costs and data interpretability by using kinetic models of ion conduction. Possible shortcomings of our approach are instead represented by the limited molecular details taken into account and the use of the diffusion theory when assessing the rate constants.

A multi-scale approach has previously been used by Bernèche and Roux (2003) to predict the current passing through the KcsA channel. Similar to what we did, they derived the energy profile encountered by the ions during permeation as well as the ion diffusion coefficient, from MD, and used this information to predict the ion movements. However, instead of using the kinetic model approach, as we did, the ion permeation was predicted by the Langevin equation that assesses the random movement of a particle along the energy profile. Interestingly, the ion energy profile was assessed by assuming a 1/3 and 2/4 K^+^ ion configuration and water between K^+^ ions, in accordance with the soft knock-on model more in vogue then. Notably, the resulting energy profile correctly predicted the concentration-current relationship at varying voltages but not the IVs that were heavily hyperlinear and distant from experimental observations.

In the same study, they found that ionic currents in the tens of picoamperes, that is, in the order of those found experimentally in KcsA channels, were obtained with energy barriers of 2‐3 kcal/mol (Bernèche and Roux, 2003). These results are in apparent contrast with ours, as we obtain similar currents with an energy barrier about twice as high. Because of this incongruence, we made a few tests on our calculation procedure. First, we checked that the rate constants we assessed using the mean first-passage time theory were compatible with the height of our energy barriers. To this end, we considered a quadratic energy barrier 5 kcal/mol high and a 2 *Å* well-to-peak distance, that is, an energy barrier similar to what we obtained for the binding/unbinding of K^+^ ions to the external sites (using a diffusion coefficient of 0.235 A^2^/ps). Using Kramer’s equation, we analytically obtained a kinetic rate constant of about 10^8^ s^−1^, that is, the same order of magnitude of our kinetic rate constants (cf. Table 1). Second, a rate of 10^8^ ions per second going through the channel pore, each carrying a charge of 1.6 10^−19^ C is expected to result in a current in the order of 10–20 pA.

Another issue that needs to be addressed in this context is why long MD simulations carried out on the KcsA channel have often resulted in predicted currents significantly smaller than those observed experimentally (Köpfer et al., 2014; Furini and Domene, 2020; Mironenko et al., 2021). Imperfect force fields, lack of an intracellular domain in the structure used in the computation, differences in the phospholipid composition, the use of periodic boundary conditions, or the method for the application of the transmembrane potential have been suggested as possible causes^1^. We would like to draw attention here to another possible cause: the size of the intracellular vestibule. K^+^ channels conductance has been suggested to heavily depend on the physical dimension of the hydrophobic intracellular vestibule (Naranjo et al., 2016). This makes it possible that the width of the intracellular vestibule of the KcsA structure chosen for MD simulations can be a major cause of the variable current predicted, due to the quite variable dimensions experimentally found in different instances (Cuello et al., 2010a; Cuello et al., 2010b). Notably, simulations using a fairly large intracellular vestibule (Cα-Cα distance at Thr112 of 32 *Å,* as derived from PDB crystal structure 3f5w (Köpfer et al., 2014)) give KcsA currents in substantial accordance with that found experimentally (up to a factor of 2). By contrast, simulations using a much smaller size for the intracellular vestibule (22 *Å,* as derived from PDB structure 5vk6 (Furini and Domene, 2020; Domene et al., 2021)) give KcsA currents about one order of magnitude smaller. Our method to calculate the current through KcsA does not include the physical dimension of the intracellular vestibule but uses a rate constant for K^+^ binding to S5 assessed by assuming K^+^ diffusion through a (large) hemispherical surface surrounding the binding site (thus minimal K^+^ diffusion restrains to reach the site). We believe that this is the reason why our modeling predicts K^+^ current amplitudes substantially higher than those found by MD and in agreement with those found experimentally. In accordance, a version of the Smoluchowski equation taking into account the diffusional restriction present in the intracellular vestibule results in a reduction in the estimated current.

We have seen that site S5, which we found right at the internal entrance of the SF, can be a major determinant of the K^+^ flux through the KcsA channel. Although site S5 is not present in the crystal structure, it has already been seen in previous MD studies of the KcsA channel. Köpfer et al. (Köpfer et al., 2014) reported K^+^ ions partially hydrated and bound to site S5, in a position very close to that found in the present study, and suggested to be an important intermediate, in conjunction with site S0, of the permeation process. MD simulations of KcsA channel permeation performed by Domene et al. (Domene et al., 2021) also show K^+^ ions sitting at a center of mass position, slightly below the tyrosine hydroxyl oxygens. Although these authors described these K^+^ ions as sitting in the cavity (at site S_cav_), their position was completely different from that shown by the crystal electron density (Morais-Cabral et al., 2001), and in fact more similar to site S5 we found in this study and also found by Köpfer et al. (Köpfer et al., 2014). A K^+^ binding site positioned just below the hydroxyl oxygen, and termed S5, was also identified with MD simulations by Jensen et al. (Jensen et al., 2010), using the Kv1.2 channel structure. Our data and the cited literature suggest that partially hydrated K^+^ ions interact with the selectivity filter at site S5 in the internal entrance that arguably represents a stable position and an important intermediate for the permeation process of the KcsA channel.

Our data could also make a small contribution to the ongoing debate between hard and soft knock-on permeation models (see (Mironenko et al., 2021) for a recent review) for a recent review. Our equilibrium results showing the stable 0/2/3/5 configuration, with sites 1 and 4 empty (0KK0 configuration), explicitly indicate that water is not present inside the SF. In fact, we never observed water molecules stably sitting in the SF at equilibrium. Although we did not conduct real-time MD simulations that could have disclosed other K^+^ configurations in the SF, our results, for what little they can say, are consistent only with the hard knock-on permeation model. We wish to add that absence of water inside the SF during K^+^ permeation was also reported by Öster et al. (Öster et al., 2019), and our 0KK0 configuration was found to be the most probable state by Domene et al. (Domene et al., 2021) and a key configuration by Köpfer et al. (Köpfer et al., 2014). Notice that the latter two studies were carried out under permeation conditions.

## Data Availability

The raw data supporting the conclusion of this article will be made available by the authors, without undue reservation.

## Footnotes

1 Incidentally, the fact that our method, essentially based on equilibrium MD computations, predicts currents close to those experimentally observed would exclude the possibility that the inconsistency derives from an erroneous force field.
