# Supplementary material for: A Multi-Scale Approach to Model K+ Permeation Through the KcsA Channel
Source: Front Mol Biosci. 2022 Jul 8;9:880660. doi: 10.3389/fmolb.2022.880660 (PMC9332843; doi:10.3389/fmolb.2022.880660)
Supplement: Supplementary file 1 [file Presentation1.pdf]

SUPPLEMENTARY

The stability of the S0/S2/S3/S5 K^+ configuration in the SF of KcsA seems to be based on fundamental physical principles, as can be seen by comparing the results of the Bikerman-PB model with classical PB model as shown in Figure S1. First, the Bikerman-PB model predicts a much lower K^+ occupancy inside the SF as compared to the classical PB model. This result is explained by the ion steric effects considered by the Bikerman-PB model, that prevents the unrealistic accumulation of K^+ ions in the SF obtained from classical PB model that treats ions as points without volume.

Saturation of K^+ ions due to steric effect, as shown in Figure S1A, strictly limits its residence inside SF and therefore $[K^+]$ is far from electrically balancing the negative charges of carbonyl oxygens. This is the reason for the ultra-low electric potential distribution at SF shown in Figure S1B. On the contrary, in the classical PB model K^+ ions are fully recruited into SF to balance the negative charges inside, and therefore a considerably higher electric potential is established, compared to the Bikerman-PB model. The severely unbalanced negative charges of carbonyl oxygens further attract K^+ ions outside the SF to reach electrostatic neutrality, where they eventually form two pileups of K^+ ions at S0 and S5, as shown in Figure S1C. This would explain why S5 near water cavity of KcsA behaves as a binding site for K^+, although electrostatic attraction is actually weak around water cavity based on the protein structure. On the contrary, if using classical PB, negative charges inside the SF are largely shielded by K^+ ions fully recruited into the SF. Therefore, pileups at S0 and S5 are obviously smaller than its counterpart by Bikerman-PB model.

While K^+ ions are strongly attracted to the SF by negative charges present there, solvation energy barrier featured by the large drop of dielectric constant as entering the SF (shown in Figure 3A) hinders K^+ ions from entering the SF due to the need of K^+ ions to dehydrate when entering the narrow SF. The result of these two competing forces, electrostatic attraction and dehydration, is near zero K^+ concentration at S1 and S4, but saturated K^+ concentration at S2 and S3. This means that when K^+ ions enter into the SF from S0 and S5, they can only occupy S2 and S3 but not S1 or S4. This almost zero residence of K^+ ions at S1 and S4 makes S1 and S4 ion depletion zones, where no ions (K^+ and Cl^-) can reside. On the contrary, as shown in Figure S1C, far over-estimated results from classical PB show that K^+ ions can reside

in all binding sites of the SF, and no ion depletion zone is present. The failure to agree with MD's 0/2/3/5 stable configuration by classical PB model again justifies the usage of Bikerman-PB model here.

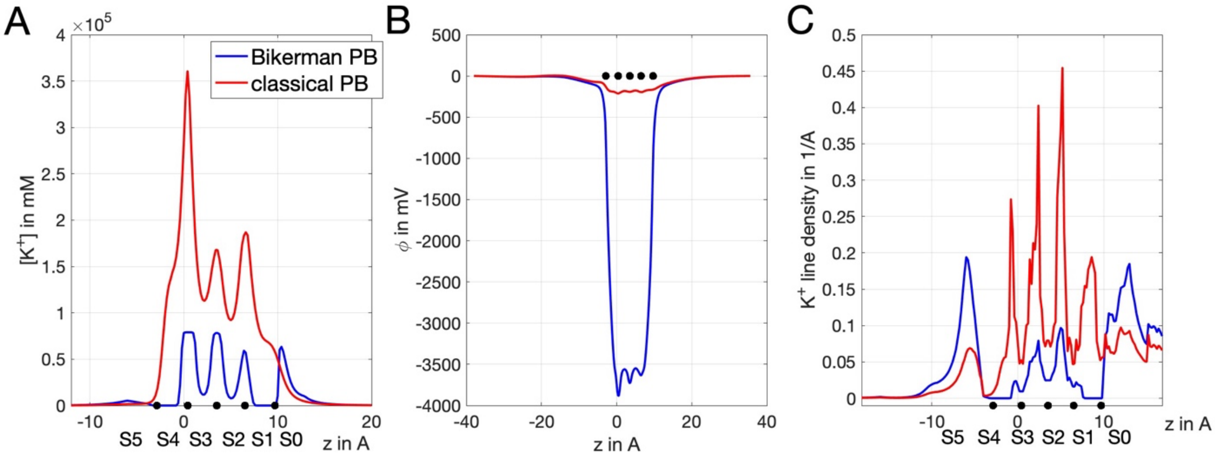

Figure S1. Comparison of Bikerman-PB and classical PB model. (A) $[K^+]$ distribution along the central axis. (B) Electric potential ϕ distribution along the central axis. (C) Line density distribution of K^+ ions along central axis. All the distributions are at equilibrium situation ($V = 0$).

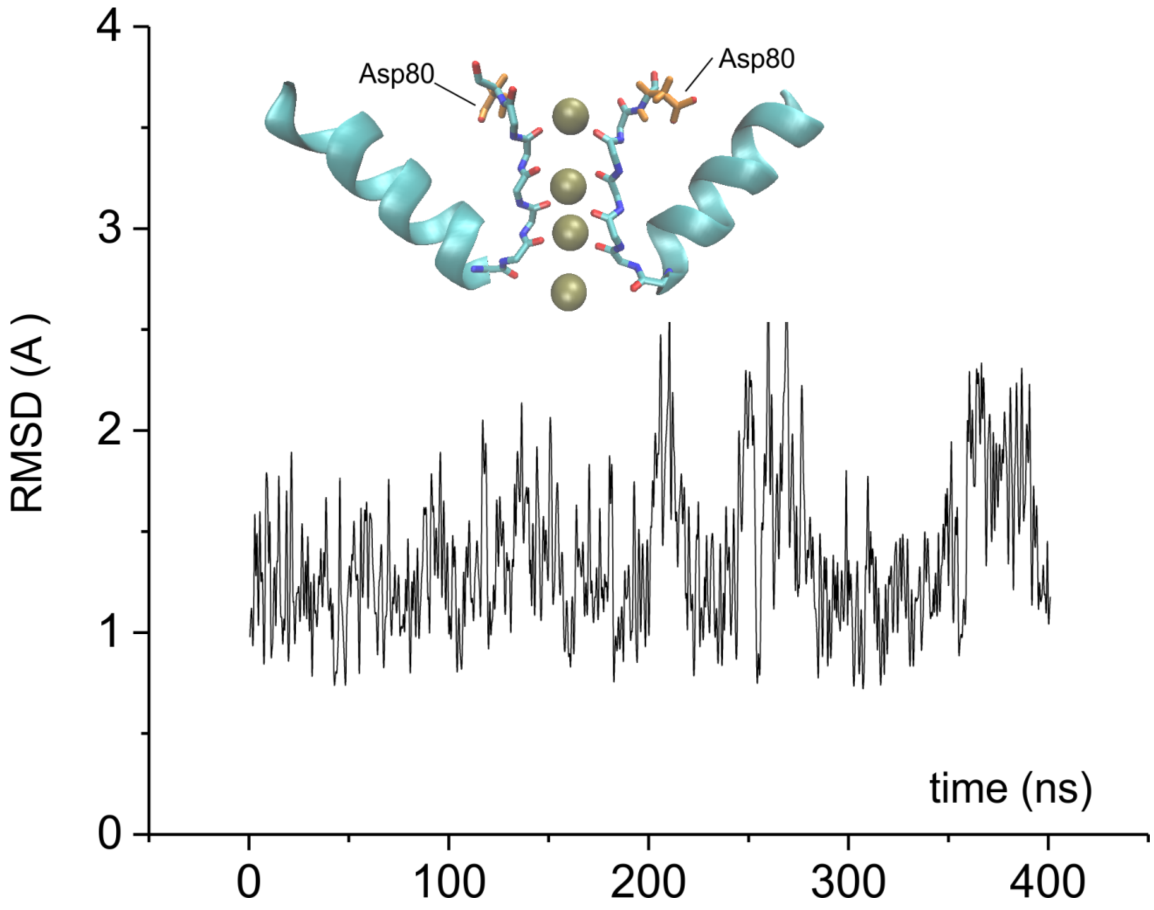

Figure S2. Plot showing the RMSD for the selectivity filter (residues 75 to 79, all atoms) in one of our MD simulations, showing that the structure appears to remain quite stable all along. The RMSD was assessed using the crystal PDB coordinates as reference. Inset: Representative structure of the selectivity filter of E71A mutant, showing the Asp80 in the non-flipped configuration as found over the entire simulation.
